# Prognostic impacts of participation in prospective surgical clinical trials on surgical outcomes in gastric cancer patients

**DOI:** 10.1038/s41598-023-42123-z

**Published:** 2023-09-09

**Authors:** Jeong Ho Song, Ho-Jung Shin, Sang-Yong Son, Hoon Hur, Sang-Uk Han

**Affiliations:** 1 Department of Surgery, Ajou University School of Medicine, 164, Worldcup-Ro, Yeongtong-Gu, Suwon, 16499 Republic of Korea; 2 Department of Surgery, Yonsei University College of Medicine, Seoul, South Korea

**Keywords:** Gastric cancer, Surgical oncology

## Abstract

Various prospective surgical trials have been conducted on treating patients with gastric cancer. In clinical practice, patients and surgeons may hesitate to participate in prospective surgical trials due to trial-related complications. In this study, we evaluated the effects of participation in prospective surgical trials on surgical outcomes after radical gastrectomy for gastric cancer. This study included 1689 patients who underwent curative gastrectomy for gastric cancer between 2016 and 2020. The propensity score weighting (PSW) method was used to adjust for differences in baseline clinicopathological characteristics between patients who participated and those who did not participate in prospective surgical clinical trials. Perioperative outcomes and overall survival were compared between groups. Of the 1689 patients, 309 (18.3%) participated in surgical clinical trials (SCT group). Before PSW, the SCT group had a similar operation time, intraoperative blood loss, complications, major complications, and hospital stay as the non-SCT group but had superior overall survival. After PSW, overall survival and perioperative outcomes were not significantly different between the groups. The present study suggests that participation in prospective surgical trials was not associated with surgical outcomes. Patients and surgeons may participate in prospective surgical trials without fearing adverse effects on surgical outcomes.

## Introduction

Since the first gastric cancer surgery by Billroth in the nineteenth century, gastric cancer treatment has evolved enormously and established itself in the current gastric cancer treatment guidelines^1–3^. The evidence-based gastric cancer treatment guidelines cited results from several pivotal randomized controlled studies, such as Dutch, MAGIC, ACTS-GC, CLASSIC, REGATTA, KLASS, CLASS, and so on^4–12^. Prospective, randomized controlled trials (RCT), such as these, are the most reliable way of comparing treatments and are considered the gold standard for studying causal relationships^13^.

In clinical practice, it is difficult for patients and surgeons to participate in prospective clinical trials, including multicenter surgical RCTs. Patients worry about treatment-related postoperative complications when receiving a new rather than existing treatment. Patients sometimes feel instinctive anxiety about the word “clinical trial.” Surgeons also feel pressured by new, unfamiliar surgical techniques, particularly when performing additional procedures for research purposes. In multicenter surgical trials, surgeons are concerned that their surgical results may be compared with those of other surgeons.

We hypothesized that participation in a prospective surgical clinical trial would not affect surgical outcomes. We aimed to evaluate the effects of participation in prospective surgical clinical trials on surgical outcomes after radical gastrectomy for gastric cancer.

## Methods

### Patients

We retrospectively reviewed a prospectively collected data from the gastric cancer database from January 2016 to December 2020 consisting of 1,915 patients who underwent gastrectomy. Patients who met one of the following criteria were excluded from the study: non-curative resection, completion of total gastrectomy, history of preoperative chemotherapy, emergency operation, or stage IV disease. Due to the retrospective nature of the study, this study was approved by the Institutional Review Board of Ajou University Hospital (AJOUIRB-DB-2023–027), and inform consent was waived.

### Surgery

All surgical procedures were performed according to the Korean and Japanese Gastric Cancer treatment guidelines^2,14^. The extent of gastric resection was determined based on tumor location. Among the patients enrolled in the SENORITA trial, which compared sentinel node navigation surgery with standard gastrectomy, those assigned to the test group underwent wedge resection of the stomach^15^. D2 lymph node dissection was performed if advanced gastric cancer or lymph node metastasis was suspected. To restore gastrointestinal continuity after surgery, we used different reconstruction methods depending on the type of gastrectomy. We employed gastroduodenostomy, gastrojejunostomy, or Roux-en-Y gastrojejunostomy for distal subtotal gastrectomy. Proximal gastrectomy was performed via double-tract reconstruction, while total gastrectomy was performed via Roux-en-Y esophagojejunostomy. Pylorus-preserving gastrectomy, on the other hand, was performed via gastrogastrostomy. The tumor stage was defined according to the 8^th^ edition of the American Joint Committee on Cancer staging system^16^. Postoperative complications were defined as any event requiring pharmacological, endoscopic, radiological, or surgical intervention until postoperative day 30. The severity of postoperative complications was graded using the Clavien–Dindo classification system^17^. Major complications were defined as Clavien–Dindo grade III or higher.

### Surgical clinical trials

Our study included patients from several prospective surgical clinical trials, collectively called the SCT group. These trials included the VIGTORY trial (n = 3), which compared D2 versus D2 plus 14v lymph node dissection in clinical T3N + and T4N + advanced gastric cancer; the AaRon trial (n = 28), which evaluated robotic distal gastrectomy for non-inferiority of N2 lymph node dissection; the ADDICT trial (n = 78), which compared D1 + versus D2 lymph node dissection in clinical stage IB or II advanced gastric cancer; the KLASS-04 trial (n = 17), which compared distal gastrectomy versus pylorus-preserving gastrectomy for middle third early gastric cancer; the KLASS-05 trial (n = 24), which compared total versus proximal gastrectomy for upper third early gastric cancer; the KLASS-06 trial (n = 34), which compared open versus laparoscopic total gastrectomy in advanced gastric cancer; the KLASS-07 trial (n = 39), which compared laparoscopy-assisted versus totally laparoscopic distal gastrectomy; the SDR-01 trial (n = 43), which evaluated the safety of sentinel lymph node-guided lymph node dissection using indocyanine green in near-infrared laparoscopy; the SDR-02 trial (n = 36), which compared quality of life and endoscopic findings according to the reconstruction methods after laparoscopic distal gastrectomy for gastric cancer; and the SENORITA trial (n = 7), which compared sentinel node biopsy with stomach-preserving surgery versus gastrectomy in early gastric cancer.

### Outcomes

The primary outcome was the development of postoperative complications according to participation in surgical clinical trials. Secondary outcomes were the development of major postoperative complications and differences in overall survival according to participation in surgical clinical trials. Overall survival was defined as the duration from surgery to death from any cause.

### Statistical analysis

The propensity score weighting (PSW) technique using a generalized boosted method (GBM) was applied to reduce differences in baseline clinicopathological characteristics between groups according to participation in surgical clinical trials. GBM was used to estimate the propensity score, and the covariates for PSW were age, sex, body mass index, American Society of Anesthesiologists physical status (ASA) score, preoperative white blood cell count, preoperative hemoglobin count, preoperative albumin count, clinical T stage, clinical N stage, operation year, operator, operation method, extent of gastric resection, extent of lymph node dissection, combined resection, tumor size, and number of harvested lymph nodes. Standardized mean difference (SMD) was calculated to assess the balance of variables between the matched groups, with an SMD < 0.1 indicating good balance and an SMD < 0.2 indicating an adequate balance between the groups^18^. Categorical variables are shown as numbers (percentages), and continuous variables are summarized as mean ± standard deviation. The chi-square and Student’s t-test were used to analyze the relationship between the groups according to participation in surgical clinical trials. The Kaplan–Meier method was used to plot the time-to-event data, and the log-rank test was used to compare group differences in time-to-event data. Statistical analyses were conducted using R software (version 3.4.4; R Foundation for Statistical Computing, Vienna, Austria). P values < 0.05 were considered statistically significant.

## Results

### Patient characteristics

**Figure 1:**
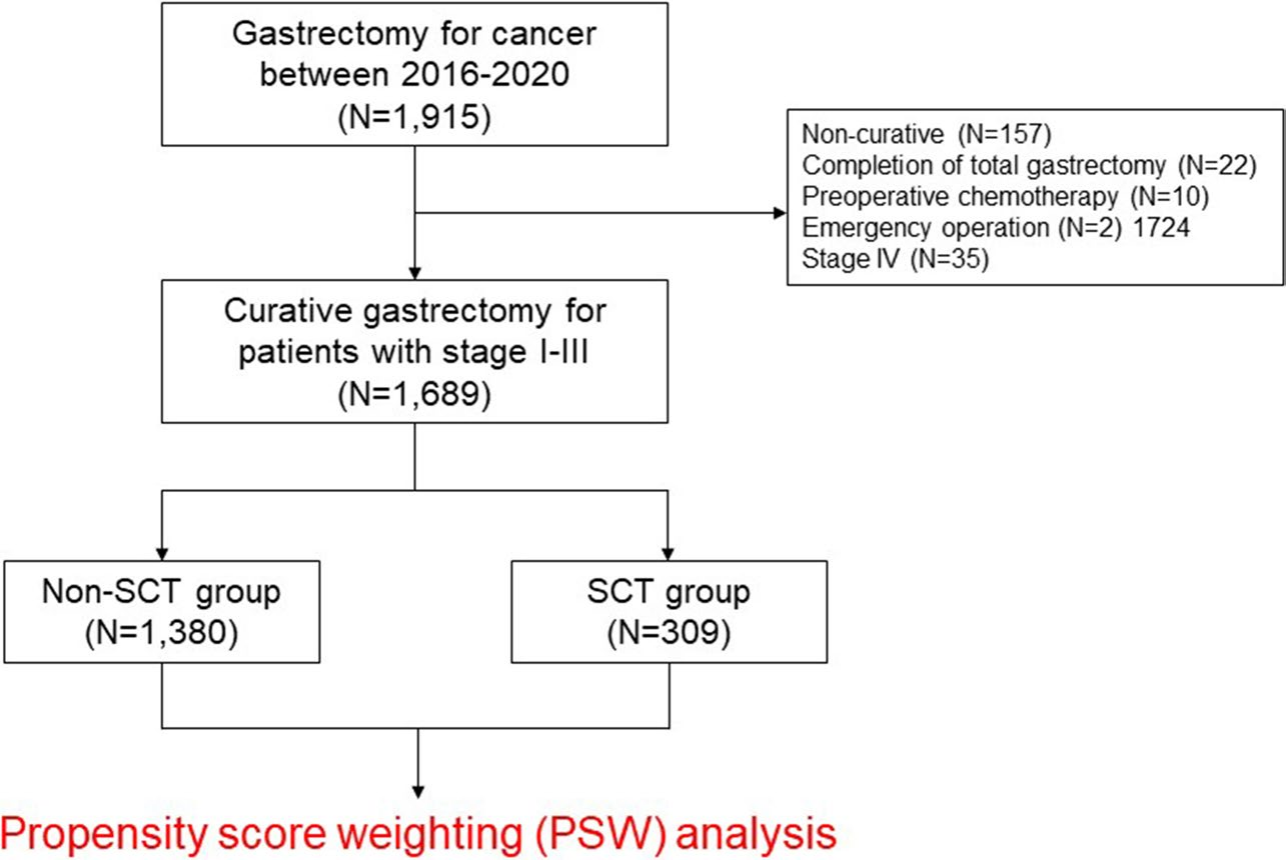
Study design.

Of the 1915 patients who underwent gastrectomy for gastric cancer, 226 were excluded from the analysis because of non-curative resection (n = 157), completion of total gastrectomy (n = 22), preoperative chemotherapy (n = 10), emergency operation (n = 2), or stage IV disease (n = 35). A total of 1689 patients were included in the study (Fig. 1).

Of the 1689 patients, 309 (18.3%) were included in the SCT group, and the remaining 1380 (81.7%) were included in the non-SCT group. The clinicopathological characteristics of the patients are summarized in Table 1. Before PSW, the SCT group was significantly younger, had a lower ASA score, higher preoperative hemoglobin and albumin counts, and earlier clinical T stage than the non-SCT group (P < 0.001, P = 0.008, P = 0.021, P < 0.001, and P < 0.001, respectively). Regarding pathologic features, the SCT group had a relatively small tumor size compared to the non-SCT group (P = 0.034). After PSW, variables that were not statistically similar before PSW were well-balanced between the groups.

**Table 1:** Patient clinicopathological characteristics.

	Unweighted population				Weighted population			
	Non-SCT group (n = 1380)	SCT group (n = 309)	P value	SMD	Non-SCT group (n = 254.84)	SCT group (n = 309)	P value	SMD
Age (years)	61.64 ± 12.46	58.84 ± 11.25	< 0.001	0.236	59.51 ± 11.45	58.84 ± 11.25	0.381	0.059
Sex								
Female	436 (31.6%)	116 (37.5%)	0.052	0.125	88.1 (34.6%)	116 (37.5%)	0.382	0.062
Male	944 (68.4%)	193 (62.5%)			166.7 (65.4%)	193 (62.5%)		
BMI (kg/m^2^)	23.96 ± 3.24	24.21 ± 3.45	0.224	0.075	24.20 ± 3.38	24.21 ± 3.45	0.987	0.001
ASA score								
I	683 (49.5%)	183 (59.2%)	0.008	0.197	139.4 (54.7%)	183 (59.2%)	0.424	0.093
II	655 (47.5%)	119 (38.5%)			108.4 (42.5%)	119 (38.5%)		
III	42 (3.0%)	7 (2.3%)			7.1 (2.8%)	7 (2.3%)		
Preoperative WBC counts	6.62 ± 1.88	6.39 ± 1.85	0.052	0.123	6.40 ± 1.78	6.39 ± 1.85	0.946	0.005
Preoperative hemoglobin level (g/dL)	13.61 ± 2.09	13.90 ± 1.62	0.021	0.157	13.94 ± 1.68	13.90 ± 1.62	0.765	0.020
Preoperative albumin level (g/dL)	4.46 ± 0.46	4.56 ± 0.32	< 0.001	0.267	4.55 ± 0.37	4.56 ± 0.32	0.455	0.048
Clinical T stage								
T1	733 (54.0%)	144 (46.8%)	< 0.001	0.366	124.9 (49.0%)	144 (46.8%)	0.625	0.094
T2	158 (11.6%)	71 (23.1%)			49.1 (19.3%)	71 (23.1%)		
T3	195 (14.4%)	55 (17.9%)			46.5 (18.3%)	55 (17.9%)		
T4	272 (20.0%)	38 (12.3%)			34.4 (13.5%)	38 (12.3%)		
Clinical N stage								
N −	926 (66.6%)	224 (72.4%)	0.059	0.125	178.3 (70.0%)	224 (72.4%)	0.426	0.056
N +	454 (33.4%)	85 (27.6%)			76.5 (30.0%)	85 (27.6%)		
Tumor size (cm)	3.67 ± 2.63	3.33 ± 2.20	0.034	0.141	3.32 ± 2.22	3.33 ± 2.20	0.947	0.004
Number of harvested LN	37.89 ± 13.35	39.04 ± 13.80	0.176	0.084	39.21 ± 13.56	39.04 ± 13.80	0.857	0.013
Lauren classification								
Diffuse	442 (32.9%)	120 (39.6%)	0.160	0.143	87.3 (34.2%)	120 (39.6%)	0.737	0.149
Intestinal	639 (47.5%)	129 (42.6%)			114.3 (44.9%)	129 (42.6%)		
Mixed	230 (17.1%)	46 (15.2%)			41.6 (16.3%)	46 (15.2%)		
Indeterminant	33 (2.5%)	8 (2.6%)			11.7 (4.6%)	8 (2.6%)		
pStage								
I	906 (65.7%)	215 (69.6%)	0.076	0.150	180.5 (70.8%)	215 (69.6%)	0.276	0.109
II	231 (16.7%)	56 (18.1%)			37.1 (14.6%)	56 (18.1%)		
III	243 (17.6%)	38 (12.3%)			37.2 (14.6%)	38 (12.3%)		

The surgical characteristics of the patients are presented in Table 2. Before PSW, there was a significant difference (P < 0.001) in the proportion of patients who had undergone recent gastrectomy between the SCT and non-SCT groups, with a higher number of patients included in the SCT group than in the non-SCT group. Two of the four surgeons actively participated in the surgical clinical trials (P < 0.001). There were statistically significant differences between the groups in the extent of gastrectomy and combined resection of other organs (P = 0.002 and P = 0.014, respectively). After PSW, all surgical variables were well balanced between the groups but showed small differences in the operator, the extent of gastrectomy, and combined resection of other organs.

**Table 2:** Patient surgical characteristics.

	Unweighted population				Weighted population			
	Non-SCT group (n = 1380)	SCT group (n = 309)	P value	SMD	Non-SCT group (n = 254.84)	SCT group (n = 309)	P value	SMD
Operation year								
2016	297 (21.5%)	42 (13.6%)	< 0.001	0.366	38.3 (15.0%)	42 (13.6%)	0.968	0.050
2017	288 (20.9%)	49 (15.9%)			38.8 (15.2%)	49 (15.9%)		
2018	292 (21.2%)	51 (16.5%)			43.1 (16.9%)	51 (16.5%)		
2019	253 (18.3%)	85 (27.5%)			70.5 (27.7%)	85 (27.5%)		
2020	250 (18.1%)	82 (26.5%)			64.1 (25.2%)	82 (26.5%)		
Operator								
A	581 (42.1%)	149 (48.2%)	< 0.001	0.376	117.1 (46.2%)	149 (48.2%)	0.145	0.131
B	515 (37.3%)	134 (43.4%)			110.0 (43.4%)	134 (43.4%)		
C	252 (18.3%)	26 (8.4%)			24.7 (9.7%)	26 (8.4%)		
D	32 (2.3%)	0			1.8 (0.7%)	0		
Operation method								
Open	109 (7.9%)	24 (7.8%)	0.410	0.087	15.9 (6.2%)	24 (7.8%)	0.560	0.075
Laparoscopy	1100 (79.7%)	255 (82.5%)			210.3 (82.5%)	255 (82.5%)		
Robot	171 (12.4%)	30 (9.7%)			28.6 (11.2%)	30 (9.7%)		
Extent of gastrectomy								
Total	258 (18.7%)	53 (17.2%)	0.002	0.200	43.6 (17.1%)	53 (17.2%)	0.144	0.187
Distal subtotal	1056 (76.5%)	232 (75.1%)			197.3 (77.4%)	232 (75.1%)		
Proximal	52 (3.8%)	12 (3.9%)			11.3 (4.4%)	12 (3.9%)		
Pylorus-preserving	8 (0.6%)	10 (3.2%)			2.1 (0.8%)	10 (3.2%)		
Wedge	6 (0.4%)	2 (0.6%)			0.6 (0.2%)	2 (0.6%)		
Lymph node dissection								
Less than D2	676 (49.0%)	1171 (55.3%)	0.050	0.127	130.1 (51.1%)	171 (55.3%)	0.221	0.086
D2 or more	704 (51.0%)	138 (44.7%)			124.7 (48.9%)	138 (44.7%)		
Combined resection	40 (2.9%)	1 (0.3%)	0.014	0.206	3.1 (1.2%)	1 (0.3%)	0.162	0.103

The perioperative outcomes of the patients are shown in Table 3. Overall, no significant differences were observed in terms of the operation time, intraoperative blood loss, complications (including details and major complications), or hospital stay between the groups (all P > 0.05). After PSW, the SCT group had a longer operation time and a higher incidence of postoperative pancreatitis. The other variables were not significantly different between the groups.

**Table 3:** Patient perioperative outcomes.

	Unweighted population				Weighted population			
	Non-SCT group (n = 1380)	SCT group (n = 309)	P value	SMD	Non-SCT group (n = 254.84)	SCT group (n = 309)	P value	SMD
Operation time	167.05 ± 48.61	165.66 ± 39.26	0.640	0.031	160.97 ± 46.75	165.75 ± 39.23	0.091	0.111
Intraoperative blood loss	96.56 ± 142.02	94.46 ± 102.61	0.990	0.001	92.45 ± 130.28	96.46 ± 102.61	0.581	0.034
Complication	277 (20.1%)	60 (19.4%)	0.856	0.016	48.8 (19.1%)	60 (19.4%)	0.919	0.007
Wound	47 (3.4%)	9 (2.9%)	0.793	0.028	8.5 (3.3%)	9 (2.9%)	0.735	0.024
Fluid collection	16 (1.2%)	7 (2.3%)	0.213	0.085	3.9 (1.5%)	7 (2.3%)	0.416	0.054
Intra-abdominal bleeding	18 (1.3%)	3 (1.0%)	0.846	0.031	2.5 (1.0%)	3 (1.0%)	0.972	0.002
Intra-luminal bleeding	18 (1.3%)	9 (2.9%)	0.074	0.112	5.1 (2.0%)	9 (2.9%)	0.392	0.058
Intestinal obstruction	23 (1.7%)	7 (2.3%)	0.630	0.043	5.6 (2.2%)	7 (2.3%)	0.951	0.004
Ileus	25 (1.8%)	3 (1.0%)	0.424	0.072	3.5 (1.4%)	3 (1.0%)	0.574	0.039
Anastomotic stenosis	6 (0.4%)	3 (1.0%)	0.461	0.064	1.0 (0.4%)	3 (1.0%)	0.212	0.068
Anastomotic leakage	16 (1.2%)	1 (0.3%)	0.310	0.098	2.5 (1.0%)	1 (0.3%)	0.267	0.082
Pancreatitis	15 (1.1%)	8 (2.6%)	0.074	0.112	2.1 (0.8%)	8 (2.6%)	0.012	0.137
Pulmonary	41 (3.0%)	8 (2.6%)	0.862	0.023	5.1 (2.0%)	8 (2.6%)	0.529	0.039
Urinary	13 (0.9%)	2 (0.6%)	0.870	0.033	2.2 (0.9%)	2 (0.6%)	0.734	0.024
Renal	7 (0.5%)	0	0.444	0.101	0.9 (0.4%)	0	0.01	0.086
Hepatic	8 (0.6%)	1 (0.3%)	0.899	0.038	1.4 (0.5%)	1 (0.3%)	0.649	0.032
Cardiac	4 (0.3%)	1 (0.3%)	> 0.999	0.006	0.3 (0.1%)	1 (0.3%)	0.385	0.042
Others	48 (3.5%)	10 (3.2%)	0.969	0.013	8.8 (3.5%)	10 (3.2%)	0.385	0.042
Major complication	89 (6.4%)	19 (6.1%)	0.947	0.012	15.5 (6.1%)	19 (6.1%)	0.970	0.003
Hospital stays	8.59 ± 4.00	8.50 ± 4.52	0.728	0.021	8.22 ± 3.21	8.50 ± 4.52	0.309	0.072

### Survival outcomes

**Figure 2:**
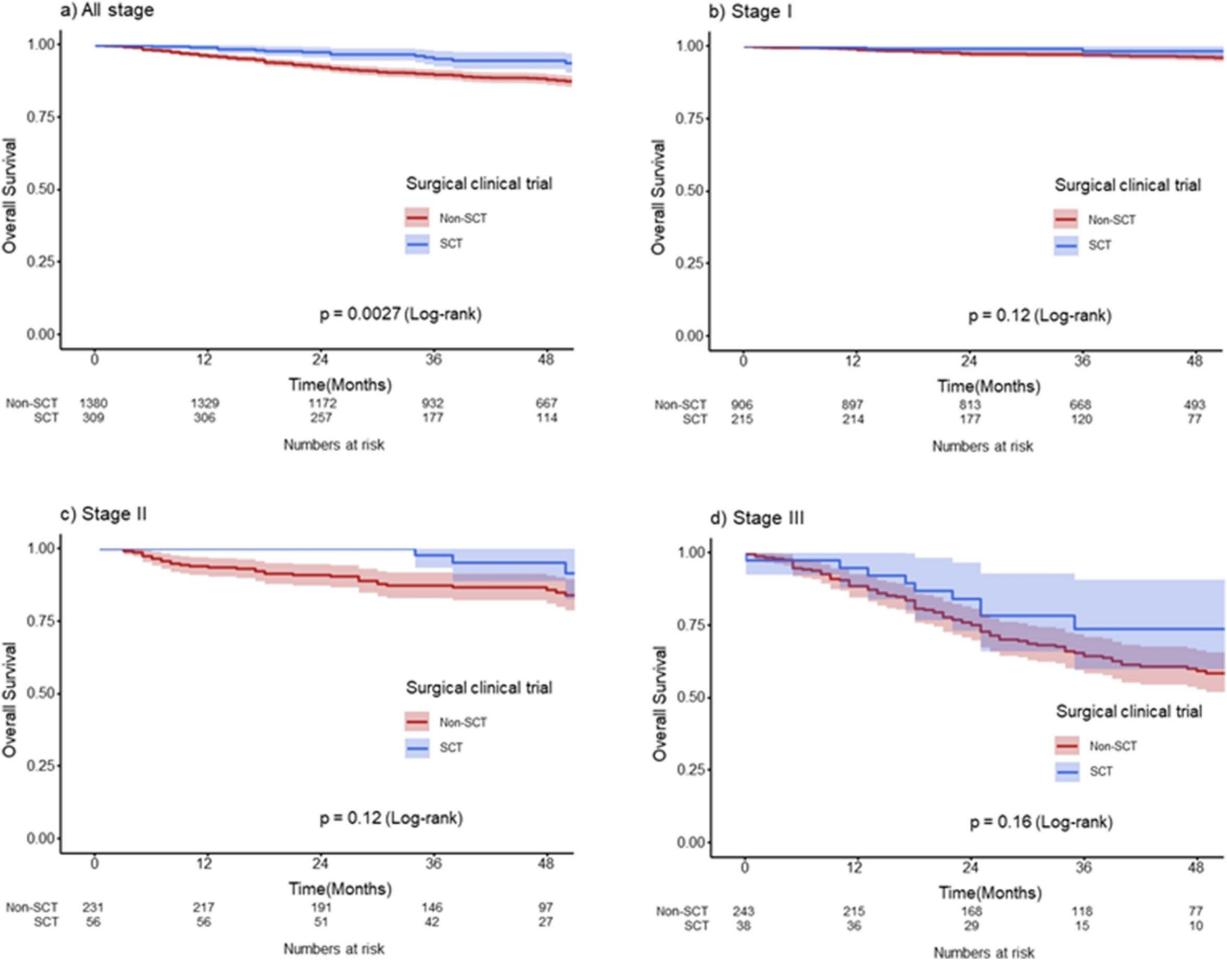
Overall survivals according to the participation in the surgical clinical trials before propensity score weighting. (**a**) All stages, (**b**) Stage I, (**c**) Stage II, (**d**) Stage III.

The median follow-up duration after gastrectomy was 48 months (range, 19–79 months) until the study cutoff date (October 2022). Of the 1689 unweighted patients, 175 (10.4%) died: 16 (9.1%) in the SCT group and 159 (90.9%) in the non-SCT group. The hazard ratio for death was 0.419 (95% confidence interval [CI], 0.247–0.712; P = 0.001) in the SCT group with reference to the non-SCT group. The 3-year overall survival rates of patients in the SCT and non-SCT groups were 95.1% and 89.6%, respectively (log-rank P = 0.003) (Fig. 2). However, the differences in 3-year overall survival were not significant when the patients were stratified according to pathologic stage (96.9% for the SCT group vs. 98.2% for the non-SCT group in stage I, P = 0.121; 87.2% for the SCT group vs. 97.7% for the non-SCT group in stage II, P = 0.123; 64.3% for the SCT group vs. 73.6% for the non-SCT group in stage III, P = 0.157).

**Figure 3:**
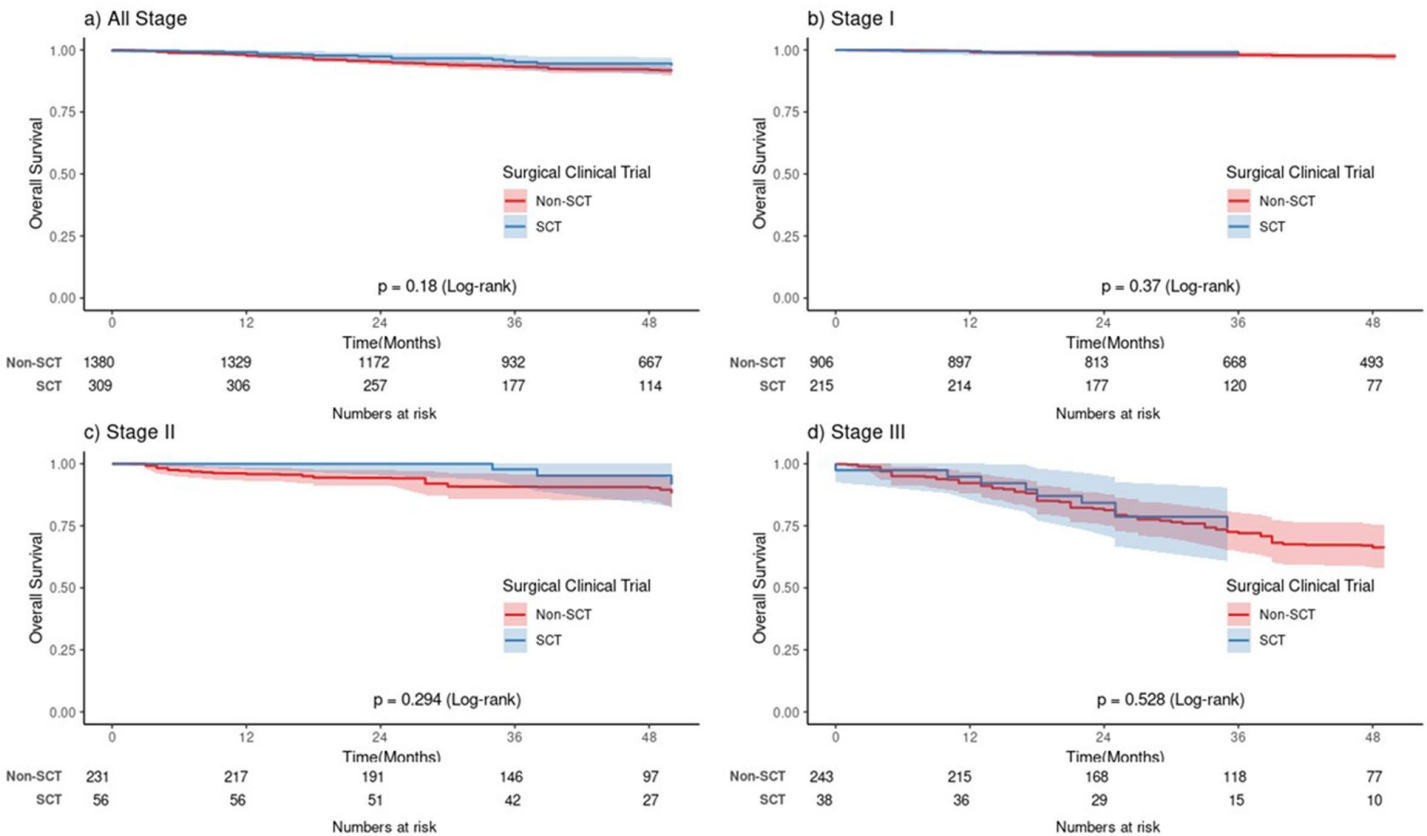
Overall survivals according to the participation in the surgical clinical trials after propensity score weighting. (**a**) All stages, (**b**) Stage I, (**c**) Stage II, (**d**) Stage III.

In the weighted populations, the hazard ratio for death in the SCT group with reference to the non-SCT group was 0.715 (95% confidence interval [CI], 0.417–1.227; P = 0.223). The 3-year overall survival rates for the SCT and non-SCT groups were 97.3% and 94.6%, respectively (P = 0.18) (Fig. 3). After stratification according to the pathologic stage, the 3-year overall survival rates of the SCT group were not significantly different from those of the non-SCT group (100% for the SCT group vs. 98.9% for the non-SCT group in stage I, P = 0.37; 100% for the SCT group vs. 95.2% for the non-SCT group in stage II, P = 0.294; 87.6% for the SCT group vs. 78.9% for the non-SCT group in stage III, P = 0.528).

## Discussion

Before being weighted with propensity scores, patients who participated in prospective surgical clinical trials had comparable postoperative morbidity and superior overall survival compared to those who did not. After weighting with propensity scores, surgical outcomes, including postoperative morbidity and overall survival, showed no differences between patients enrolled in surgical clinical trials and those who did not.

Even surgeons familiar with prospective surgical clinical trials sometimes feel nervous when performing surgeries on patients registered for clinical trials. In particular, when operating on patients requiring additional procedures for research purposes in addition to standard surgical procedures, surgeons may experience tension and be overwhelmed by unfamiliar procedures. Even when surgical clinical trials are actively conducted, patient safety is of utmost importance. Therefore, whether participation in a prospective surgical clinical trial per se impacts surgical outcomes is a topic that needs to be clarified.

In recent decades, several prospective surgical clinical trials have been conducted worldwide. The Republic of Korea is one of the leading countries in gastric cancer-related surgery and research. Our institution, a major gastric cancer center in Korea, is committed to enrolling patients in several prospective trials. In this study, the number of patients enrolled in surgical clinical trials among all patients undergoing gastric cancer surgery increased over time, reaching nearly 25% (82/250) by 2020. Ten surgical clinical trials were performed during the five years of the study period. Of these, seven were RCTs and the other three were prospective non-randomized surgical trials. The prospective trials required additional procedures besides standard surgery, which may have made the surgeon feel nervous or overwhelmed. As a result, these cases were included in the SCT group.

In the unweighted populations, the proportion of patients who underwent surgical clinical trials in the entire cohort was over 18%. The patients in the SCT group had few known risk factors for postoperative complications. They had a younger age, lower ASA score, higher preoperative hemoglobin and albumin levels, smaller tumor size, and earlier tumor stages^19–21^. This reflects the bias of researchers who recommend surgical clinical trial participation for a relatively healthy and limited number of patients with far advanced gastric cancer. The perioperative outcomes of the SCT group in the unweighted population were similar to those of the non-SCT group, despite having fewer risk factors for postoperative complications. This suggests a possible worsening of perioperative outcomes in the SCT group; however, the two groups had similar outcomes in the weighted population. The overall survival in the SCT group was superior to that in the non-SCT group. However, this superiority may be due to discrepancies in clinicopathological features between the groups, and the task of matching the discrepancies was performed using the PSW method.

RCTs are the best method for analyzing causal relationships, but only a retrospective analysis was possible in this study because it was designed to classify patients according to their participation in prospective studies. Enrolling patients for this study prospectively meant that all patients were allocated to the SCT group. A good alternative for correcting the clinicopathological background of the two groups in a retrospective study was the use of propensity scores. We tried to add as many matching variables as possible to adjust for the two groups evenly and introduced the PSW method to minimize the number of patients who were excluded from the control group.

After PSW, the perioperative outcomes, including each complication type, were comparable between the groups, except for pancreatitis and renal complications. As the incidence of these complications was low, further validation is needed in a study targeting a larger number of patients. The overall survival of the SCT group, which was superior before PSW, was comparable to that of the non-SCT group after PSW. Prospective studies assume that participation in a surgical clinical trial has no significant impact on surgical outcomes, including operation time, intraoperative blood loss, complications, hospital stay, and oncological outcomes. However, to the best of our knowledge, this has not been validated in well-designed studies. This study is expected to encourage patients and surgeons to participate more actively in prospective studies by providing relief to those concerned about adversely affecting the surgical results through participation in a surgical clinical trial.

This study had several limitations. First, since our study was conducted at a single institution, selection bias is possible despite our efforts to reduce it using propensity weighting. Second, long-term recurrence- or relapse-free survival could not be analyzed because of the lack of recurrence-related data. Additional research is needed on adjuvant chemotherapy and recurrence, depending on the participation in clinical trials.

In conclusion, enrolling relatively healthy patients with early tumor lesions was associated with participation in surgical clinical trials. Performing PSW to match the clinicopathological features between the groups, participation in surgical clinical trials did not affect surgical outcomes, including postoperative complications and long-term overall survival. Participation in well-designed surgical clinical trials for medical advancement should be actively encouraged by patients and surgeons without worrying about surgical outcomes.

## Data Availability

The data that support the findings of this study are available on request from the corresponding author.
